# Increased Growth-Inhibitory and Cytotoxic Activity of Arsenic Trioxide in Head and Neck Carcinoma Cells with Functional p53 Deficiency and Resistance to EGFR Blockade

**DOI:** 10.1371/journal.pone.0098867

**Published:** 2014-06-13

**Authors:** Mariya Boyko-Fabian, Franziska Niehr, Luitpold Distel, Volker Budach, Ingeborg Tinhofer

**Affiliations:** 1 Translational Radiooncology Laboratory, Department of Radiooncology and Radiotherapy, Charité University Hospital, Berlin, Germany; 2 Department of Radiation Oncology, University Erlangen-Nuremberg, Erlangen, Germany; INSERM-Université Paris-Sud, France

## Abstract

**Background and Purpose:**

Mutations in the *p53* gene are frequently observed in squamous cell carcinoma of the head and neck region (SCCHN) and have been associated with drug resistance. The potential of arsenic trioxide (ATO) for treatment of p53-deficient tumor cells and those with acquired resistance to cisplatin and cetuximab was determined.

**Material and Methods:**

In a panel of 10 SCCHN cell lines expressing either wildtype p53, mutated p53 or which lacked p53 by deletion the interference of p53 deficiency with the growth-inhibitory and radiosensitizing potential of ATO was determined. The causal relationship between p53 deficiency and ATO sensitivity was evaluated by reconstitution of wildtype p53 in p53-deficient SCCHN cells. Interference of ATO treatment with cell cycle, DNA repair and apoptosis and its efficacy in cells with acquired resistance to cisplatin and cetuximab was evaluated.

**Results:**

Functional rather than structural defects in the *p53* gene predisposed tumor cells to increased sensitivity to ATO. Reconstitution of wt p53 in p53-deficient SCCHN cells rendered them less sensitive to ATO treatment. Combination of ATO with irradiation inhibited clonogenic growth in an additive manner. The inhibitory effect of ATO in p53-deficient tumor cells was mainly associated with DNA damage, G2/M arrest, upregulation of TRAIL (tumor necrosis factor-related apoptosis-inducing ligand) receptors and apoptosis. Increased activity of ATO was observed in cetuximab-resistant SCCHN cells whereas cisplatin resistance was associated with cross-resistance to ATO.

**Conclusions:**

Addition of ATO to treatment regimens for p53-deficient SCCHN and tumor recurrence after cetuximab-containing regimens might represent an attractive strategy in SCCHN.

## Introduction

Arsenic trioxide (ATO) which has been used for more than 2,000 years in Chinese traditional medicine for treatment of almost every disease has made a remarkable comeback into classical medicine after its high efficacy for treatment of acute promyelocytic leukemia (APL), reported by Chinese doctors, had been confirmed by the results from randomized clinical trials in Europe and the United States [1]–[3]. The impressive complete remission and survival rates observed in APL prompted the subsequent testing of ATO also in other neoplastic diseases. These studies revealed that besides specifically targeting the promyelocytic leukemia gene product (PML) and the APL-specific fusion protein of PML with the retinoic acid receptor alpha (PML-RAR-a) thereby promoting cell differentiation of leukemia cells, ATO can interfere with mitochondrial functions, the cellular redox system, the cell cycle and apoptosis. Since these cellular functions are generally involved in the response of tumor cells to ionizing radiation the radiosensitizing efficacy of ATO was subsequently evaluated. The first report of a synergistic activity of ATO in combination with radiotherapy came from a murine solid tumor model [4] and these early promising results were subsequently confirmed in xenograft models of glioma [5], [6], fibrosarcoma [7], cervical cancer [8] and oral squamous cell carcinoma [9]. Of note, despite its radiosensitizing activity in tumor tissue the addition of ATO to radiotherapy did not result in a significant increase in normal tissue toxicity [4], [9].

As predictive biomarker for enhanced pro-apoptotic and growth-inhibitory activity of ATO structural defects in the *p53* gene have originally been described in models of B-cell lymphoma [10] and multiple myeloma [11], [12] which could also explain the low toxicity profile in normal cells expressing wildtype (wt) p53. Since p53 mutations occur very frequently in SCCHN and have been linked to shorter overall survival [13], increased risk of local recurrence [14], [15] and radioresistance [16] the combination of radiotherapy with ATO might represent a novel promising therapeutic strategy in SCCHN. To address this question we evaluated in the present study whether p53 deficiency might be predictive for increased cytotoxic and growth-inhibitory activity of ATO in SCCHN cells. The effects of ATO alone and its combination with irradiation (IR) on clonogenic survival, cell cycle progression and apoptosis were evaluated in a panel of p53-deficient and -proficient SCCHN cell lines. Since ATO treatment has also been shown to activate the EGFR pathway [17], to interfere with surface EGFR expression levels [18] and to modulate EGFR-mediated DNA double-strand break repair [19] we also assessed the growth-inhibitory activity of ATO in a SCCHN cell line model of acquired cetuximab resistance. In addition, potential cross-resistance between ATO and cisplatin was evaluated.

## Material and Methods

### Cell lines and reagents

The previously established SCCHN cell lines SCC9 [20], UD (University of Düsseldorf) -SCC-2, -4, -5 [21], UT (University of Turku) -SCC-9 [22], UM (University of Michigan) -SCC-11B, -17B, -25 and -74B [23] were kindly provided by T.K. Hoffmann (University of Essen, Dept. of Otorhinolaryngology) and T.E. Carey (University of Michigan, Head and Neck Cancer Biology Laboratory). The SCCHN cell line FaDu was purchased from ATCC. The identity of the cell lines was confirmed by high-throughput SNP-based authentication (Multiplexion, Heidelberg, Germany). All cell lines were tested for mycoplasma at monthly intervals by RT-PCR [24]. Contaminated cultures were treated with Mycoplasma Removal Agent (MP Biomedicals, Santa Ana, USA) according to the manufacturer's protocol.

All cell lines with the exception of UD-SCC-2 were HPV-negative. A detailed review on general characteristics and molecular features of these cell lines has been previously published by Lin and coworkers [25]. Two cell line models of acquired resistance to cisplatin and cetuximab were established by treating FaDu and UT-SCC-9 with increasing doses of cisplatin or cetuximab, respectively, for a period of 4 to 8 months.

Cells were cultured in Minimal Essential Medium (MEM) supplemented with 15% heat-inactivated fetal bovine serum and 1X non-essential amino acids. All cell culture reagents were from Gibco (Invitrogen, Darmstadt, Germany). Cell cultures were incubated at 37°C and 5% CO_2_ in a humidified atmosphere. Arsenic trioxide (ATO) was purchased from Sigma-Aldrich (Munich, Germany). It was dissolved in 1 M sodium hydroxide (NaOH) solution to generate a 25 mM-solution which was further diluted in H_2_O to generate a 1 mM-stock solution. Working solutions were freshly prepared from the stock solution by dilution in cell culture medium on the day of the experiment. Cetuximab was provided by Merck Pharma GmbH (Darmstadt, Germany). Cisplatin was purchased from Sigma-Aldrich.

### Molecular analysis of the p53 genotype

The previously reported gene sequence of *p53* within the coding region of the SCCHN cell lines was confirmed by sequencing the full-length transcripts after their PCR amplification. Total cellular RNA extraction was performed using the High-Pure RNA Isolation kit (Roche Diagnostics, Mannheim, Germany). Synthesis of cDNA was done with the ‘Omniscript Reverse Transcription kit’ (QIAGEN, Hilden, Germany) according to the supplied protocol using random hexamers and oligo dT15 primers (Roche, Basel, Switzerland) and 2 µg of total RNA.

PCR was carried out in a reaction volume of 25 µl containing 2 µl cDNA, 2.5 µl 10× PCR buffer, 2.0 mM MgCl_2_, 100 nM of each primer, the four deoxynucleoside triphosphates (200 µM each) and 1 unit of InviTaq DNA polymerase (Invitek GmbH, Berlin, Germany). For amplification of the whole coding region two primer pairs were used: forward primer 1: 5′-CTTCCGGGTCACTGCC-3′; reverse primer 1: 5′ GCTGTGACTGCTTGTAGATG-3′, amplifying a 518-bp fragment of the p53 cDNA; forward primer 2: 5′-GTTGATTCCACACCCCCGCCC-3′; reverse primer 2: 5′-GTGGGAGGCTGTCAGTGGGGA-3′ amplifying a PCR product of 782 bp in length. PCR cycling was carried out on a thermal cycler (Eppendorf, Hamburg, Germany). After initial denaturation at 95°C for 5 min, the reaction was carried out at 95°C denaturation for 1 min, 50°C (primer 1)/66°C annealing (primer 2) for 30 s, and 72°C elongation for 90 s for 45 cycles. The extension was lengthened to 5 min for the last cycle. PCR products were stained with Sybr Green and analyzed by agarose gel electrophoresis. After purification using the Qiaex II Gel Extraction kit (Qiagen) samples were sent to Source Bioscience (Berlin, Germany) for sequencing. For the cell lines lacking or expressing too low levels of p53 mRNA direct dideoxynucleotide sequencing of all *p53* exons was performed.

### p53 transcriptional activity assay

As read-out for p53 transcriptional activity in SCCHN cell lines, basal and irradiation-induced p21 expression levels were determined by quantitative reverse-transcriptase polymerase chain reaction (qRT-PCR). Total cellular RNA extraction was performed using the High-Pure RNA Isolation kit (Roche Diagnostics, Mannheim, Germany). Synthesis of cDNA was done with the ‘Omniscript Reverse Transcription kit’ (QIAGEN, Hilden, Germany) according to the supplied protocol using random hexamers and oligo dT15 primers (Roche, Basel, Switzerland) and 2 µg of total RNA.

The quality of RNA was checked by GAPDH PCR and only samples positive for GAPDH transcripts were used for analysis. Realtime PCR was performed in a reaction volume of 20 µl containing 2 µl cDNA, Light Cycler TaqMan Master (Roche), primers and probes for p21 and the housekeeping gene porphobilinogen deaminase (PBGD) in concentrations recommended by the manufacturer (Real Time Ready Assays, Roche). PCR cycling was performed using the Light Cycler 480 II (Roche). Relative quantification of p21 expression was done by normalization to the expression levels of PBGD using the ΔC_t_-method.

### Clonogenic survival assays

Cells were seeded into 12-well plates at a density of 300 cells/well. Twenty four hours after seeding, cells were left untreated or were treated with increasing doses of ATO, irradiation (IR) or the combination of both. Irradiation was performed using a 250 kV deep x-ray unit (Philips RT250) and single doses up to 6 Gy were applied. Non-irradiated cultures were processed along with irradiated cultures. If not stated otherwise, cells were incubated with ATO 2 hs before irradiation for combined treatment. Cells were then incubated for up to 14 days. At the end of the experiments colonies were fixed, stained using 10% Giemsa stain solution and colonies containing >50 cells were counted. Plating efficiency and survival fractions for given treatments were calculated on the basis of survival of non-treated cells. All samples were done in triplicates and at least three independent experiments were carried out. The median inhibitory concentration (IC50) of ATO and the combination index (CI) for the combined treatment with ATO and IR were calculated using the CalcuSyn software version 2.1 (Biosoft, Cambridge, UK).

### MTT cell viability assay

The 3-(4,5-dimethylthiazol-2-yl)-2,5-diphenyltetrazolium bromide (MTT) assay was performed in order to determine the sensitivity of cisplatin-resistant FaDu cells (FaDu_CDDP-R_) and cetuximab-resistant UT-SCC-9 cells (UT-SCC-9_CET-R_) as well as their parental sensitive counterparts to ATO treatment. Briefly, cells were seeded in 96-well plates. After 24 hs, ATO was added and cells were incubated for up to 10 days. Cell viability was assessed by measuring the absorbance of the formazan solution. Each sample was analyzed in six technical replicates and the experimental series were repeated for four times. Survival fractions were calculated on the basis of untreated cells.

### Reconstitution of wt p53 in SCC9 cells

SCC9 cells were stably transfected with the tetracycline-inducible expression vector pRTS1 [26], either encoding for wt p53 (SCC9-wtp53) or as an empty vector (SCC9-vc). Briefly, plasmid DNA (20 µg) was transfected into 10^6^ cells by electroporation in 400 µl Optimem (Invitrogen) at 250 V and 975 µF using a Biorad electroporation apparatus. Immediately after electroporation, cells were resuspended in MEM growth medium supplemented with 10% FCS and were allowed to recover for 48 hs at 37°C and 5% CO2. Selection of transfected cells was performed by their subsequent cultivation for 4 weeks in complete growth medium additionally supplemented with hygromycin B (Calbiochem) to a final concentration of 70 µg/ml. For activation of conditional gene expression of wt p53, cells were treated with doxycycline (Dox) at the indicated concentrations.

### Immunoblotting

The expression levels of p53 and its downstream target p21 in SCC9-wtp53 and SCC9-vc cells were assessed by standard immunoblotting. Briefly, cells were treated with doxycycline as indicated in the figure legends. Standard SDS-polyacrylamide gel electrophoresis was performed using 60 µg of total protein per cell lysate, followed by transfer to PVDF membranes (EMD Millipore, Billerica, MA, US). The following antibodies were used for detection: mouse anti-human p53 (clone DO-1, Santa Cruz, Santa Cruz, CA, USA), mouse anti-human p21 (clone Ab-1, Calbiochem, EMD Millipore Corporation) and peroxidase-conjugated goat anti-mouse IgG (Jackson ImmunoResearch Laboratories, West Grove, PA, USA). The immunoreactivity was detected using the ECL plus Western Blot detection system (Amersham Biosciences, GE Healthcare Europe, Freiburg, Germany).

### Detection of apoptosis by assessing annexin-V-FITC and propidium iodide

Cells were seeded into 12-well plates at a cell density of 3×10^4^ cells/well. Twenty four hours later, cells were treated for 96 hs with ATO, IR and the combination of both at defined concentrations and doses. At the end of the experiment, cells were harvested by trypsinization and washed in three subsequent washing steps with culture medium, phosphate-buffered saline (PBS) and annexin-V binding buffer (ABB, 10 mM HEPES/NaOH, pH 7.4; 140 mM NaCl; 2.5 mM CaCl_2_). Phosphatidylserine on the outer leaflet of the plasma membrane as specific marker of apoptotic cells was detected by staining of cells in ABB containing annexin-V labeled with fluorescein isothiocyanate (FITC) at a concentration recommended by the manufacturer (Alexis Biochemicals, ENZO Life Sciences, Exeter, United Kingdom). For the discrimination of apoptotic and necrotic cells, the cell-membrane impermeable dye propidium iodide (PI, final concentration: 1 µg/ml) was added to the staining solution. After staining for 15 minutes, cells were immediately analyzed using a FACSCanto II cytometer (BD Biosciences Europe, Heidelberg, Germany). At least, 10,000 events were recorded. Data analysis was performed with BD FACSDiva Software v6 (BD Biosciences).

### Assessment of cell cycle distribution

Cells were harvested by trypsinization, washed and re-suspended in 0.5 ml PBS. Fixation was performed by drop-wise addition of an equal volume of ice-cold ethanol. After washing with PBS cells were re-suspended in 0.5 ml propidium iodide (PI) staining solution (20 µg/ml PI, 0.1% (v/v) Triton-X, 200 µg/ml DNAse-free RNAse in PBS). Samples were stored overnight at 4°C and analyzed on the next day. The relative number of cells in the G0/G1 and G2/M phases of the cell cycle and the number of apoptotic cells with DNA fragmentation (sub-G1 peak) were determined by flow cytometry.

### Determination of surface TRAIL receptors and nuclear gamma-H2AX by flow cytometry

Cells were incubated with different concentrations of ATO for 4 to 48 hs, harvested by trypsinization and fixed by ethanol. Cells were incubated with antibodies in staining buffer (1% bovine serum albumin and 0.2% (v/v) Triton-X in PBS) for 20 min and were subsequently analyzed using the FACSCanto II cytometer. The following antibodies were used for staining: mouse anti-TRAIL-R1 (clone DJR1) and TRAIL-R2 (clone DJR2-4, both PE-labeled, eBioscience, Hatfield, UK); mouse anti-p-H2AX (clone JBW301, Merck-Millipore, Darmstadt, Germany), and rabbit anti-mouse Alexa Fluor 488 (Life Technologies, Darmstadt, Germany).

### Statistical analysis

All statistical analyses were performed using StatView Software (SAS Institute Inc., Version 5.0.1). The significance of differences in the distribution of cells in the cell cycle and the extent of apoptosis were determined using the paired t-test. The differences in clonogenic survival after treatment of p53-deficient and -proficient cell lines with ATO were evaluated for significance using the ANOVA unpaired t-test. The level of significance was set at p<0.05.

## Results

### The p53 functional status of SCCHN cells correlates with their sensitivity to ATO treatment

**Figure 1:**
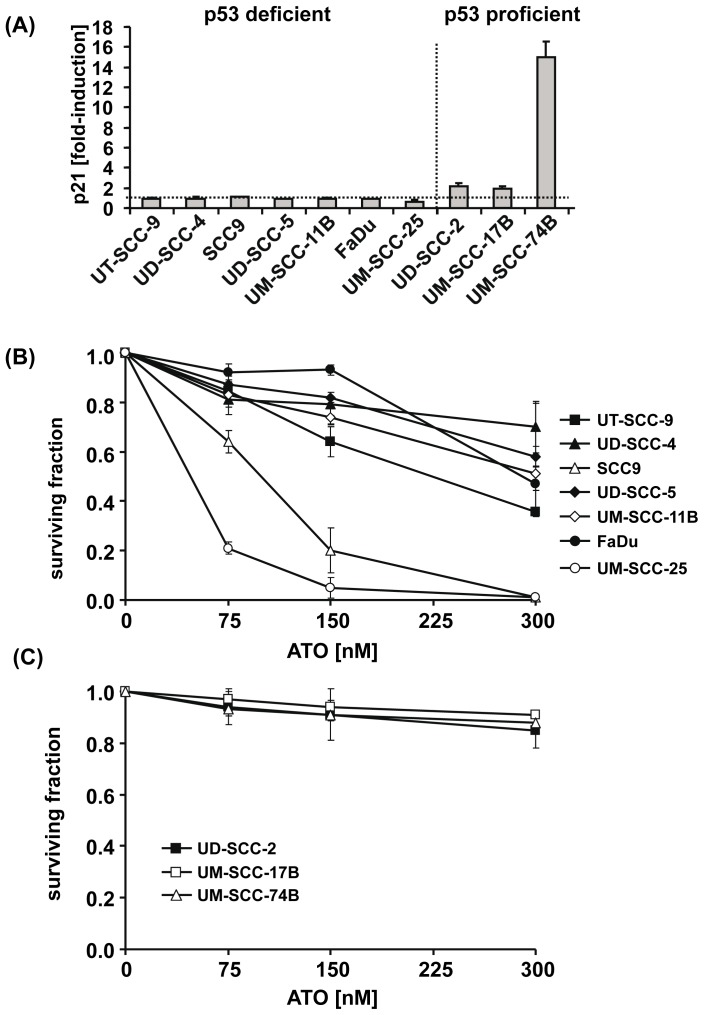
The p53 status interferes with the growth-inhibitory activity of ATO. (A) Cells were left untreated or were irradiated with a single dose of 6 Gy. Four hours after IR, cells were harvested and their expression levels of p21 as a functional read-out for p53 transcriptional activity were determined by qRT-PCR. Relative quantification of p21 expression was done by normalization to the expression levels of porphobilinogen deaminase (PBGD) and to the untreated control using the ΔΔC_t_-method. IR-induced p21 expression (mean fold induction and standard error) is presented. The p53-deficient and p53-proficient cell lines were grouped using >1.5-fold induction of p21 by IR as threshold. (B, C) Cells were seeded at a density of 300 cells/well in 12-well plates and incubated for a period of 10–14 days in the absence or presence of the indicated doses of ATO. Survival fractions for given treatments were calculated on the basis of the survival of non-treated cells. Each sample was done in triplicate. The results from at least three independent experiments with p53-deficient (B) and p53-proficient cell lines (C) are presented. The symbols for each individual cell line are given in the graphs.

In order to determine whether the p53 status interferes with the cytotoxic and growth-inhibitory activity of ATO we first confirmed the previously reported *TP53* genotype of the SCCHN cell lines by sequencing (Table 1). In addition, we assessed the p53 transcriptional activity in each cell line using IR-induced expression of the p53 target gene p21 as a functional read-out [27]. The p21 expression levels before and 4 hs after IR of cells with a single dose of 6 Gy were quantified by qRT-PCR. This analysis revealed the absence of any p21 upregulation in cell lines with deletion or mutation in the *TP53* gene while a 2- to 15-fold induction was observed in cell lines with wt p53 (Figure 1 A, Table 1). Although no genetic lesion in the complete coding sequence of *p53* could be detected we failed to observe any significant IR-induced induction of p21 in UM-SCC-25 cells (Table 1). We therefore allocated this cell line to the p53-deficient group (Figure 1 A).

**Table 1:** IC50 values and combinatory index for treatment of p53-proficient and p53-deficient SCCHN cell lines with ATO and IR.

Cell line	p53 genotype	Class of mutation	p21 fold induction*	IC50 [μM ATO]	CI at ED75 (IR+ATO)	Effect
UT-SCC-9	Δ exon 2-9	disruptive	1.0	0.21	1.08	additive
UD-SCC-4	c.449 del13	frameshift	1.0	2.41	0.86	additive
SCC9	c.820 del32	frameshift	1.1	0.09	1.29	antagonism
UD-SCC-5	H179Y	missense	1.0	0.45	0.95	additive
UM-SCC-11B	C242S	missense	1.0	0.31	0.98	additive
FaDu	R248L	missense	0.9	0.36	1.04	additive
UM-SCC-25	wt	–	0.7	0.03	0.69	synergism
UD-SCC-2	wt	–	2.1	3.3	0.90	additive
UM-SCC-17B	wt	–	2.0	4.5	1.00	additive
UM-SCC-74B	wt	–	15.0	31.9	0.93	additive

*IR-induced p21 induction as a measure of p53 function was determined 4 hs after irradiation of cells with 6 Gy.

Subsequently, we compared the effect of ATO treatment on clonogenic survival in the group of p53-deficient and p53-proficient SCCHN cell lines. As seen in Figure 1 B, treatment of p53-deficient SCCHN cells with submicromolar doses (75–300 nM) of ATO reduced their clonogenic survival in a dose-dependent manner. In SCCHN cell lines expressing functional p53 the same doses of ATO only slightly interfered with their clonogenic survival (Figure 1 C). The mean inhibition of clonogenic survival at 300 nM of ATO was 62% in the p53-deficient group (range: 30% to 90%) and 12% (range: 9% to 15%) in the p53-proficient group which was significantly different (ANOVA unpaired t-test, p = .015). The inhibitory effect of ATO on clonogenic survival in the p53-deficient group was independent of whether the genetic lesion in the *TP53* gene consisted of a deletion of exons 2–9 leading to a p53-null phenotype (UT-SCC-9), a frameshift mutation leading to truncated p53 protein (UD-SCC-4, SCC9) or a single missense mutation within the DNA-binding domain of p53 (UD-SCC-5, UM-SCC-11B, FaDu) and was also observed in cells lacking any genomic lesion within the *TP53* coding sequence but expressing p53 transcripts without transactivation activity (UM-SCC-25).

**Figure 2:**
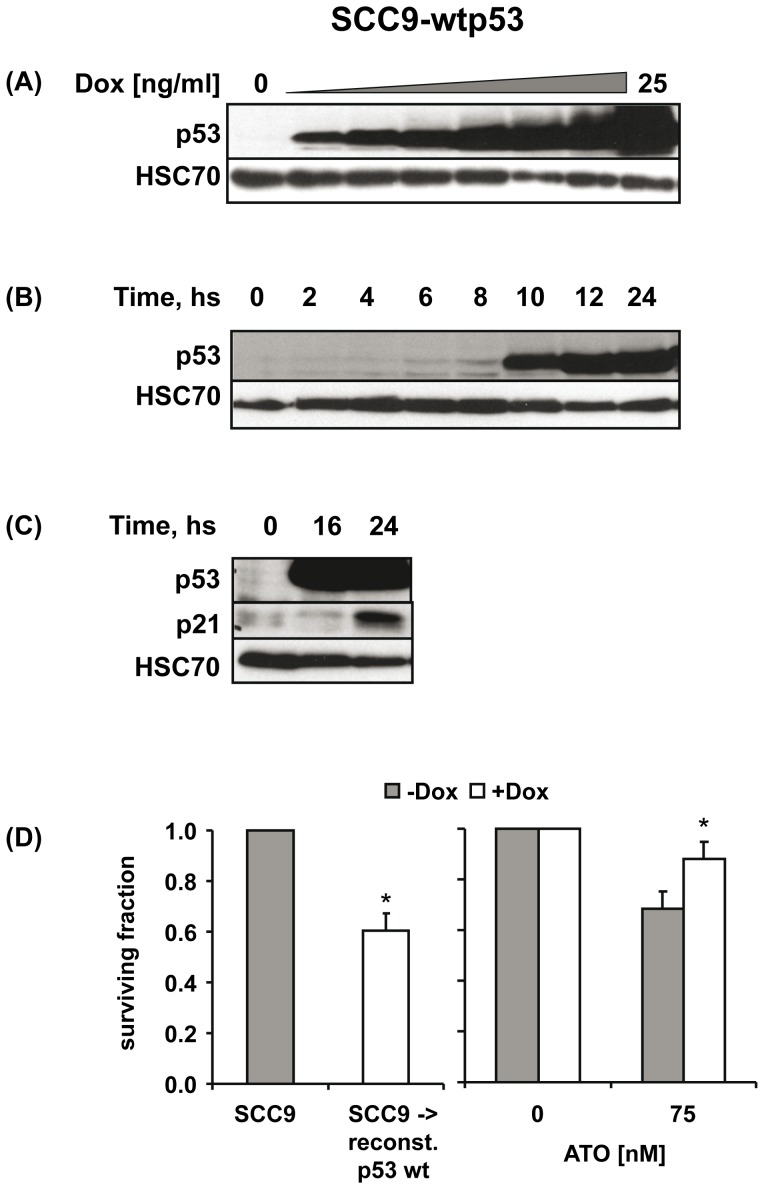
Reconstitution of p53-deficient SCC9 cells with wt p53 renders them less sensitive to ATO treatment. (A) SCC9-wtp53 cells were treated with increasing doses of Dox for 24 hs or (B) with a dose of 20 ng/ml Dox for the indicated time periods. The expression of p53 was detected by immunoblotting. Detection of HSC70 served as protein loading control. (C) Induction of p53 by treatment of SCC9-wtp53 cells with 20 ng/ml Dox was followed by upregulation of p21. (D) Reconstitution of wt p53 after Dox treatment inhibited the clonogenic growth of SCC9 cells (left graph). After correction for this growth-inhibitory effect of wt p53 itself, a significantly reduced sensitivity of SCC9-wtp53 cells to ATO treatment (75 nM) was observed (right graph). * p<0.05 (paired t-test).

In order to establish a causal relationship between p53 deficiency and increased sensitivity of SCCHN cells to ATO we assessed whether reconstitution of wt p53 in p53-deficient SCC9 cells would decrease their sensitivity to ATO treatment. SCC9 cells were stably transfected with the tetracycline-inducible expression vector pRTS1 [26], either encoding for wt p53 (SCC9-wtp53) or as an empty vector (SCC9-vc). In the absence of doxycycline (Dox), expression of p53 and p21 was not detectable in SCC9-wtp53 cells. Addition of Dox led to a significant induction of wt p53 expression in a dose- (Figure 2 A) and time-dependent manner (Figure 2 B) which preceded upregulation of its target gene p21 (Figure 2 C). No induction of p53 and p21 was observed after Dox treatment of SCC9-vc cells (data not shown). We then evaluated the effect of ATO on the clonogenic survival of SCC9-wtp53 cells in the absence (-Dox) or presence of wt p53 (+Dox). Reconstitution of wt p53 decreased clonogenic survival of SCC9-wtp53 cells (Figure 2 D left panel). After correcting for this growth-inhibitory effect of wt p53 reconstitution itself we observed a significantly reduced sensitivity of these cells to treatment with ATO at low doses (Figure 2 D right panel).

### Combined treatment of SCCHN cells with ATO and IR inhibits their clonogenic survival in an additive manner

**Figure 3:**
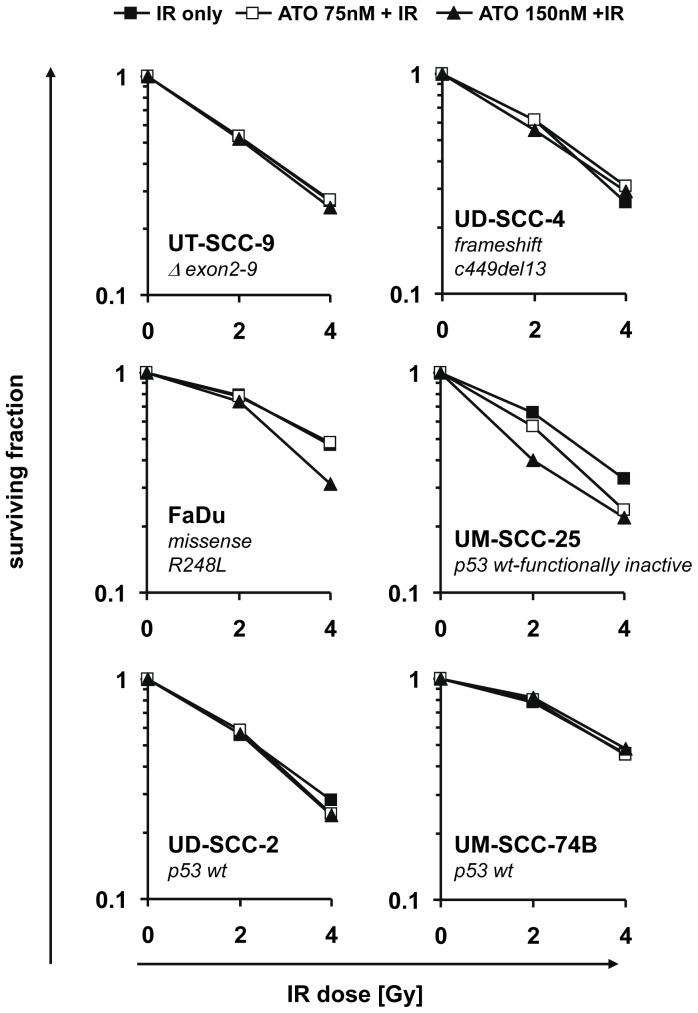
ATO combined with IR inhibits the clonogenic survival of SCCHN cells in an additive manner. Cells were seeded at a density of 300/well in 12-well plates. Twenty four hours after seeding, cells were left untreated or were treated with the indicated doses of ATO, IR or the combination of both. Non-irradiated cultures were processed along with irradiated cultures. Cells were incubated for up to 14 days. Survival fractions for given treatments were calculated on the basis of survival of non-treated cells and corrected for the effect of ATO alone. Each sample was done in triplicate. Mean values from at least three independent experiments are presented. The symbols for the different treatments are given in the top of the figure. The *p53* genotype of each individual cell line is depicted in the graphs.

Based on previous reports of a significant radiosensitizing activity of ATO in a xenograft model of oral squamous cell carcinoma [9] we next asked whether we could observe such effect in our SCCHN cell lines as well and whether the radiosensitizing activity of ATO would also depend on the p53 status. When cells were treated with ATO 2 hs before IR their clonogenic survival was inhibited more effectively than by either treatment alone. After correction for the cytotoxic activity of ATO itself no significant radiosensitizing activity, neither in p53-deficient nor p53-proficient SCCHN cell lines with the exception of the UM-SCC-25 cell line, could be observed (Figure 3). The calculated combinatory indices (Table 1) indeed suggested an additive but not synergistic effect of the combination regimen in 9 of 10 cell lines. Since there is evidence from previous studies that the interaction between ATO and IR could depend on the sequence of their combination we also treated cells with ATO 2 hs after IR. Again, only additive effects of the combined treatment were observed (data not shown).

### Cell cycle arrest, residual DNA double strand breaks and apoptosis by ATO in p53-deficient SCCHN cells

**Figure 4:**
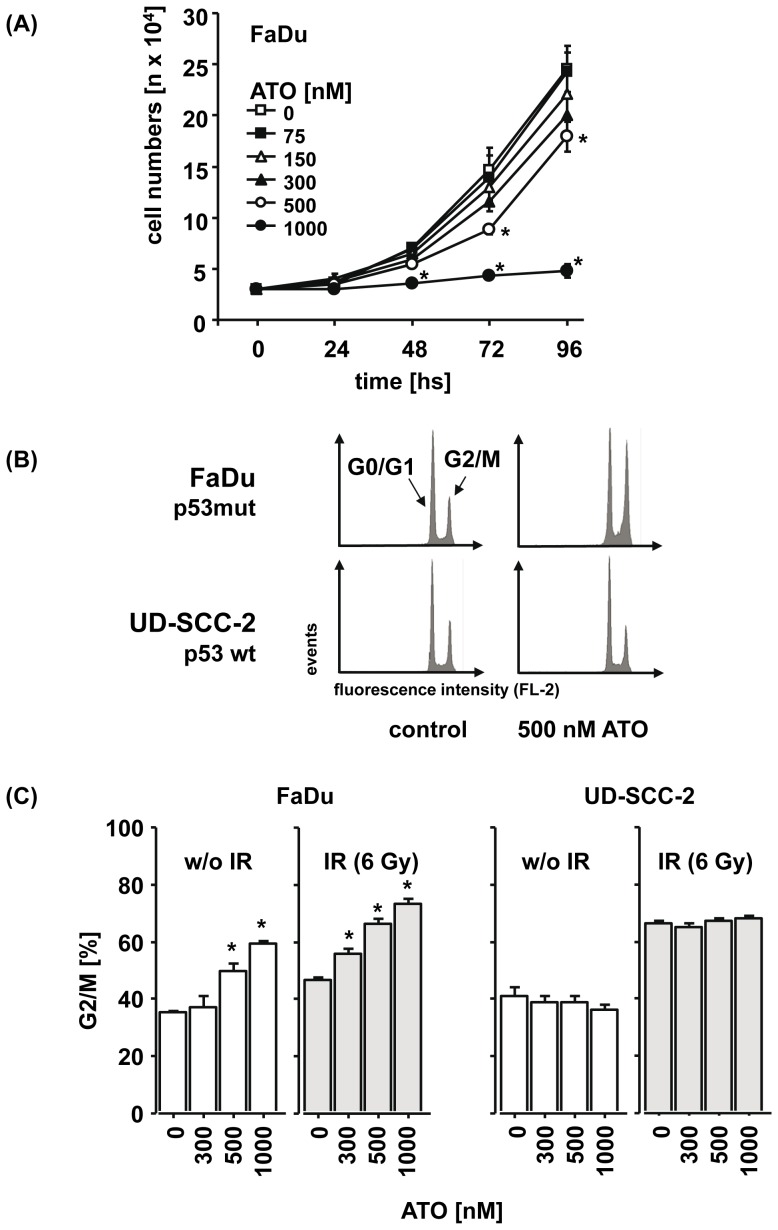
The growth-inhibitory effect of ATO in p53-deficient FaDu cells depends on dose and time and is associated with a cell cycle arrest in G2/M. (A) FaDu cells were left untreated or were treated with increasing doses of ATO. After the indicated time, cells were harvested by trypsinization and cell numbers were counted. The mean cell numbers ± SEM of three independent experiments are presented. (B) Representative flow cytometry histograms of the cell cycle distribution are presented which were observed in p53-deficient FaDu (upper panel) and p53-proficient UD-SCC-2 cells (lower panel), either untreated (left) or after treatment with 500 nM ATO (right). (C) After treatment of FaDu cells and UD-SCC-2 cells with ATO, irradiation or the combination of both, cells were harvested and the relative numbers of cells in G2/M were determined by flow cytometry. The mean percentages ± SEM are presented. Asterisks mark samples for which significant differences compared to the untreated control (p<0.05) were observed.

Considering the manifold interactions of ATO with diverse cellular functions [28] we were then interested in its mechanisms of action in SCCHN cells lacking functional p53. In order to characterize direct effects of the drug we chose shorter incubation times than in the clonogenic survival assays and tested a broader concentration range of ATO in this set of experiments. Using FaDu cells as a model for p53-deficient SCCHN cells, we first determined the influence of ATO on cell proliferation. As shown in Figure 4 A, the exponential increase in cell numbers over time was significantly inhibited by ATO at a concentration of 500 nM and completely blocked by 1 µM of ATO. A comparable dose-dependent inhibitory activity could also be observed when the metabolic activity of FaDu cells was determined using the MTT assay (data not shown).

**Figure 5:**
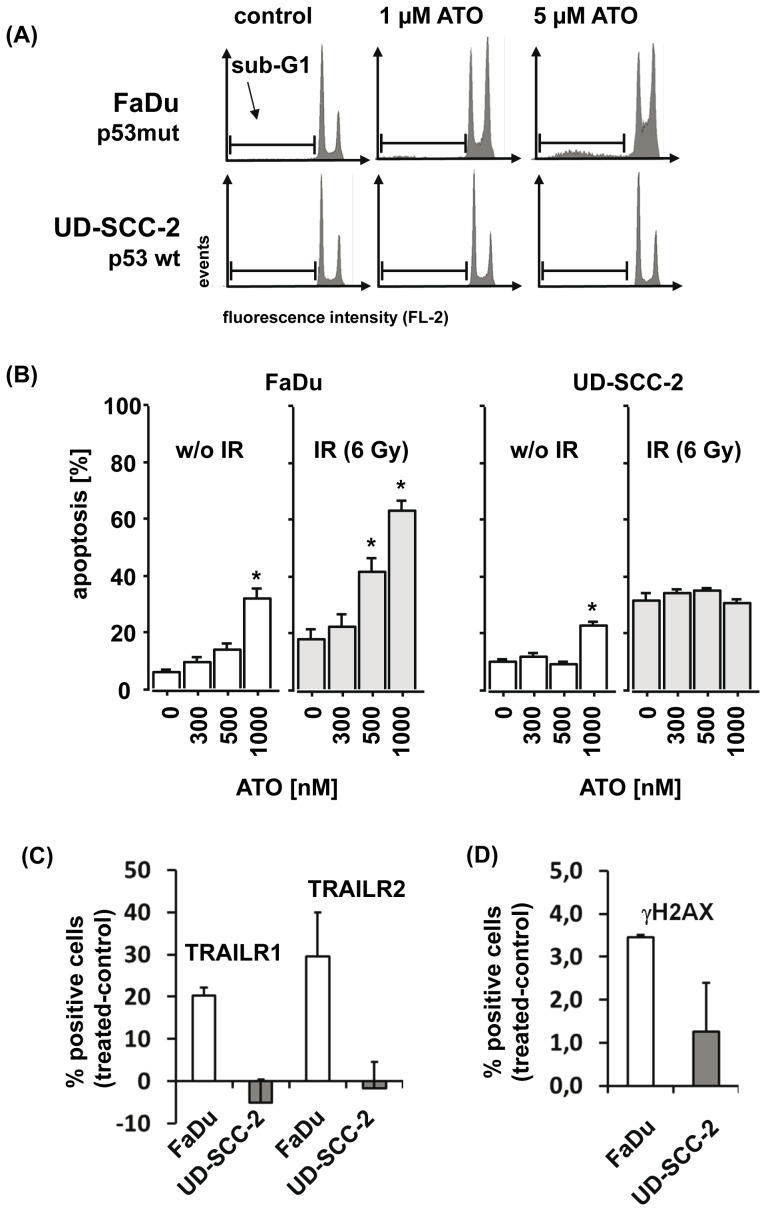
ATO induces apoptosis in p53-deficient FaDu but not p53-proficient UD-SCC-2 cells. (A) Representative flow cytometry histograms of sub-G1 analysis after treatment of p53-deficient FaDu (upper panel) and p53-proficient UD-SCC-2 cells with the indicated doses of ATO are presented. (B) FaDu and UD-SCC-2 cells were left untreated or were treated with ATO for 96 hs at the indicated doses. The mean percentages of cells with features of apoptosis, detected by annexinV-FITC/PI staining and flow cytometry analysis are presented. *significant differences compared to untreated control (p<0.05). (C, D) FaDu and UD-SCC-2 cells were left untreated or were treated with 5 µM of ATO for 48 hs. The relative changes in the mean percentages of cells expressing TRAILR1 and TRAILR2 (C) or displaying residual DNA double strand breaks, as determined by gamma-H2AX staining (D) are presented.

We further assessed whether the observed inhibition of cell proliferation was mediated by a blockade in cell cycle progression or was a result of direct induction of apoptosis. After treatment with ATO for 96 hs we observed a dose-dependent arrest in the G2/M phase of the cell cycle in the p53-deficient FaDu but not in p53-proficient UD-SCC-2 cells (Figure 4 B, C). In line with the results from the clonogenic survival assay, the combination of ATO with IR increased the inhibitory effect on cell cycle progression in an additive manner in the p53-deficient but not -proficient cells (Figure 4 C). Beside the effect on cell cycle direct induction of apoptosis by ATO alone (Figure 5 A) and in combination with IR (Figure 5 B) was observed and again, the p53-deficient FaDu cells were significantly more sensitive to the pro-apoptotic activity of ATO than the p53-proficient UD-SCC-2 cells.

Increased cytotoxic activity of ATO has previously been linked with increased activation of the extrinsic cell death program via TRAIL receptors [11], [12], [29] and reduced capability of tumor cells to repair DNA double strand breaks [19]. In order to assess any potential interference of ATO with these cellular programs in SCCHN cells, we treated p53-deficient (FaDu) and proficient cells (UD-SCC-2) with ATO for 48 hs. We then evaluated any potential changes in their surface expression of TRAIL-R1 and TRAIL-R2 by flow cytometry. No basal expression of TRAIL-R1 and TRAIL-R2 was found in any of the two cell lines. After treatment with ATO the expression of both death receptors was induced in the p53-deficient but not in the proficient cell line (Figure 5 C). Assessment of nuclear gamma-H2AX as a specific marker for DNA double strand breaks revealed reduced repair capacity of the p53-deficient compared to the p53-proficient cell line (Figure 5 D).

### The growth-inhibitory effect of ATO in SCCHN cells with acquired cetuximab and cisplatin resistance

**Figure 6:**
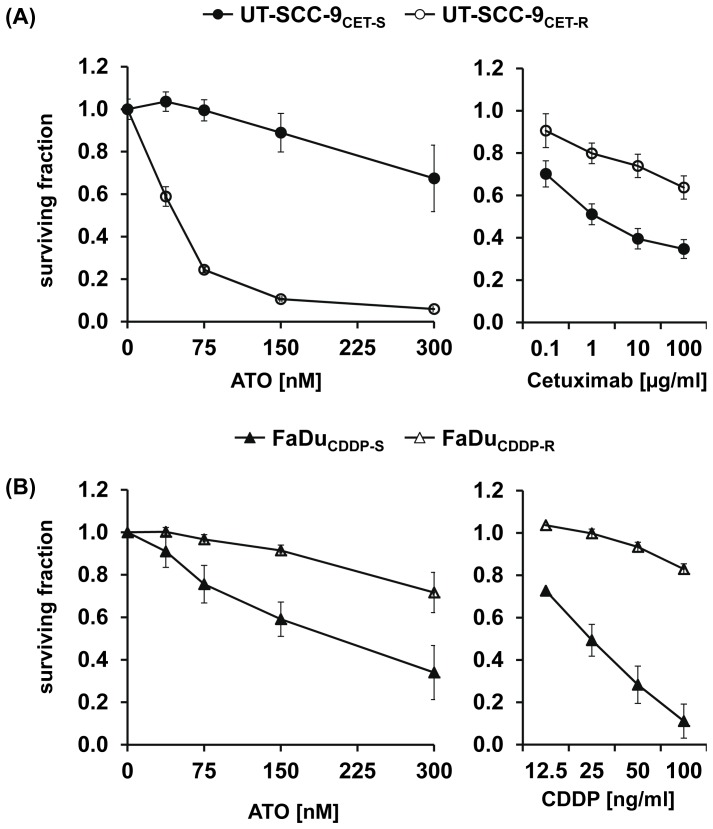
CDDP-resistant SCCHN cells show cross-resistance to ATO whereas cetuximab-resistant cells display increased ATO sensitivity. The sensitivity of (A) cetuximab-resistant UT-SCC-9 cells (UT-SCC-9_CET-R_) to ATO (left panel) or cetuximab (right panel) compared to their parental sensitive counterparts (UT-SCC-9_CET-S_) was determined by the MTT assay. In addition, (B) the sensitivity of cisplatin-resistant FaDu cells (FaDu_CDDP-R_) and parental FaDu_CDDP-S_ to ATO or CDDP treatment was determined. Briefly, cells were treated for 10 days with the drugs at the indicated concentrations. Cell viability was assessed by measuring the absorbance of the formazan solution. Each sample was analyzed in six technical replicates and the experimental series were repeated for four times. Survival fractions were calculated on the basis of untreated cells. The mean surviving fractions ± SEM are presented.

Cisplatin (CDDP) and cetuximab are the two major components of concurrent radiochemotherapy for first-line treatment of primary SCCHN. Since the 5-year recurrence rates after radiochemotherapy are still considerably high and since treatment most probably selects for tumor cells with resistance to the respective agents, we evaluated the potential of ATO for treatment of recurrent disease in two SCCHN models of acquired resistance to CDDP and cetuximab. These models had been established by long-term treatment with increasing concentrations of these drugs. Assessment of viability using the MTT assay after long-term drug treatment revealed that the phenotype of acquired resistance was stable up to a minimum of 6 months after stopping the selection process by removing the drug from the cultures. A significant difference in the sensitivity of resistant subclones (UT-SCC-9_CET-R_, FaDu_CDDP-R_) compared to the parental cells (UT-SCC-9_CET-S_, FaDu_CDDP-S_) could be observed (Figures 6 A, B, right panels). In the model of acquired cetuximab resistance we observed a significant and pronounced increase in the sensitivity of cetuximab-resistant SCCHN cells to ATO treatment (Figure 6 A). In contrast, CDDP-resistant FaDu_CDDP-R_ cells were cross-resistant to ATO treatment (Figure 6 B).

## Discussion

In this study, we could demonstrate that ATO at doses below the clinically achieved plasma levels of current ATO-containing treatment regimens in APL [30] displayed significant growth-inhibitory and cytotoxic activity preferentially in p53-deficient SCCHN cells and increased the inhibitory effect of ionizing radiation on clonogenic survival in an additive manner. The addition of ATO to current treatment regimens could thus represent a potential treatment strategy to improve the therapeutic outcome of SCCHN patients with p53-deficient tumors.

Although mutations within the *TP53* gene are considered the most frequent [31], [32] and one of the earliest genetic alterations [33], [34] in the carcinogenesis of SCCHN their prognostic value is still a matter of debate. This is mainly due to the small number of patients, the lack of a focus on a particular tumor site and the methodological differences in the assessment of *TP53* mutations in the majority of the published studies so far precluding a conclusive meta-analysis [35]. Nonetheless, there is accumulating evidence that patients presenting with tumors harboring disruptive [13], truncating [15] or loss-of-function mutations in the *TP53* gene [36] belong to a group of patients with poor prognosis and increased risk of treatment failure [13], [15], [16], [36]. *TP53* mutations are highly enriched in patient cohorts of HPV-negative carcinomas [37]. Results from large clinical trials revealed impaired efficacy of all components of the state-of-the-art SCCHN treatment regimes in patients with HPV-negative carcinomas and this has been linked at least in part with p53 deficiency [38]. These patients could potentially benefit from novel combinatory regimens including ATO.

In our cell line model, we observed an additive but not synergistic interaction between ATO and IR. This was not surprising since former *in vivo* studies could already demonstrate that the radiosensitizing effect of ATO is mainly based on its interaction with the tumor microenvironment and not a direct modulation of the cellular radiosensitivity of tumor cells [4], [7]. In murine xenograft models, an immediate vascular shutdown followed by extensive central necrosis after a single application of ATO has been reported [4]. This antivascular effect of ATO was accompanied by an increase in the intratumoral levels of the known vasoactive mediator TNF-α [4] and was associated with an increased therapeutic efficacy of radiotherapy. Comparable results were reported for fibrosarcoma xenografts in which the vascular-damaging activity of ATO was mainly observed in regions of low pH and poor oxygenation [7], conditions inherent to tumor sites. These features of ATO together with its preferential activity in p53-deficient SCCHN cells reported here strongly suggest a favorable therapeutic window for the combination of ATO and IR in p53-deficient tumors.

The molecular basis for the preferential sensitivity of p53-deficient tumor cells to ATO treatment remains largely elusive. Distinct effects of ATO on G1 and G2 cell cycle checkpoints leading to differences in the extent and duration of the G2/M arrest and the induction of mitotic arrest-associated apoptosis in p53-deficient and p53-proficient cells have previously been reported for the model of multiple myeloma [11] and the Li-Fraumeni syndrome [39] and comparable results were obtained from studies of other DNA-damaging agents such as paclitaxel [40]. In line with these reports we observed a more pronounced G2/M arrest and increased relative numbers of apoptotic cells in p53-deficient compared to p53-proficient SCCHN cell lines. We also confirmed the previously reported upregulation of TRAIL receptors [11], [12], [29] and accumulation of DNA double strand breaks after ATO treatment [19]. As for the G2/M arrest, these molecular changes were more pronounced in p53-deficient SCCHN cells. A more detailed analysis of changes in the expression or activation status of regulators of cell cycle, apoptosis and DNA repair will be necessary to reveal the molecular determinants of increased ATO sensitivity in p53-deficient SCCHN cells.

We could demonstrate that assessment of p53 functional activity enabled a better prediction of ATO sensitivity than the p53 mutational status. However, the integration of the analysis of IR-induced p21 expression in the clinical algorithm of individual treatment selection seems difficult given that tumor tissue harvested before and after the first radiation would have to be analyzed. As an alternative, vital tissue sections prepared from surgical specimens of SCCHN patients might be more suitable for *ex vivo* assessment of p53 functionality and identification of ATO-sensitive tumors before starting treatment. A pilot study for the evaluation of p53 functionality as predictive pretreatment biomarker as opposed to sole assessment of HPV status has been initiated in our laboratory.

The analysis of our cell line models of acquired drug resistance revealed that cetuximab resistance was associated with increased while CDDP resistance was correlated with decreased sensitivity to ATO. This interesting observation certainly deserves confirmation in additional models of cetuximab resistance. Transient activation of the EGFR signaling pathway in normal and tumor cells upon exposure to arsenic has been reported in several studies [17], [19], [41]–[43]. This cellular response has been shown to antagonize the ATO-induced apoptotic response, thereby contributing to the insensitivity of solid tumors to ATO treatment [17], [19], [42]. Cetuximab resistance which is characterized by similar molecular features, such as increased activation of the downstream effector kinases Src, PI3K and AKT in the EGFR signaling pathway, should therefore rather be linked to ATO resistance. Future studies will be needed to elucidate the molecular basis for the unexpected contrary correlation in our study.

Our results of a cross-resistance between CDDP and ATO in SCCHN cells are in line with the results from a previous study in bladder cancer [44]. As one potential mechanism, upregulation of protective anti-oxidative enzymes which has been associated with CDDP resistance [45], [46] as well as resistance to arsenicals [47] might be involved in the observed cross-resistance.

In conclusion, we identified ATO as potentially valuable drug for treatment of p53-deficient SCCHN, recommending its further evaluation for HPV-negative SCCHN given the high frequency of *TP53* loss-of-function mutations in this patient subset. Its previously reported synergistic activity with radiotherapy *in vivo* strongly supports preclinical and phase I clinical evaluation of this treatment combination in the future.
